# Early Identification and Intervention of Schizophrenia: Insight From Hypotheses of Glutamate Dysfunction and Oxidative Stress

**DOI:** 10.3389/fpsyt.2019.00093

**Published:** 2019-02-27

**Authors:** Chieh-Hsin Lin, Hsien-Yuan Lane

**Affiliations:** ^1^Department of Psychiatry, Kaohsiung Chang Gung Memorial Hospital, Chang Gung University College of Medicine, Kaohsiung, Taiwan; ^2^School of Medicine, Chang Gung University, Taoyuan, Taiwan; ^3^Graduate Institute of Biomedical Sciences, China Medical University, Taichung, Taiwan; ^4^Department of Psychiatry and Brain Disease Research Center, China Medical University Hospital, Taichung, Taiwan; ^5^Department of Psychology, College of Medical and Health Sciences, Asia University, Taichung, Taiwan

**Keywords:** glutamate, N-methyl-D-aspartate receptor, oxidative stress, early psychosis, schizophrenia, prodrome, biomarker

## Abstract

Schizophrenia is a severe mental disorder which leads to functional deterioration. Early detection and intervention are vital for better prognosis. However, the diagnosis of schizophrenia still depends on clinical observation to date. Without reliable biomarkers, schizophrenia is difficult to detect in its early phase. Further, there is no approved medication for prodromal schizophrenia because current antipsychotics fail to show satisfactory efficacy and safety. Therefore, to develop an effective early diagnostic and therapeutic approach for schizophrenia, especially in its prodromal phase, is crucial. Glutamate signaling dysfunction and dysregulation of oxidative stress have been considered to play important roles in schizophrenic prodrome. The N-methyl-D-aspartate receptor (NMDAR) is one of three types of ionotropic glutamate receptors. In this article, we reviewed literature regarding NMDAR hypofunction, oxidative stress, and the linkage between both in prodromal schizophrenia. The efficacy of NMDAR enhancers such as D-amino acid oxidase inhibitor was addressed. Finally, we highlighted potential biomarkers related to NMDAR and oxidative stress regulation, and therefore suggested the strategies of early detection and intervention of prodromal schizophrenia. Future larger-scale studies combining biomarkers and novel drug development for early psychosis are warranted.

## Introduction

Schizophrenia is a high-morbidity and high-mortality brain disorder. Globally 1% population suffered from this disorder. The common symptoms of schizophrenic patients include hallucination, delusion, disorganized thought and behavior, and negative symptoms. Clinical manifestation of schizophrenia consists of three domains: positive symptoms (such as hallucinations or delusions), negative symptoms (such as flattening affect or social withdrawal), and cognitive deficits (such as impaired memory, attention, and executive functions) (1–4). Among them, cognitive function impairments are considered to be core symptoms of schizophrenia, starting from its prodromal phase, while psychotic symptoms have not yet been vivid (5–9). Cognitive deterioration appears at an earlier age in schizophrenia patients (10, 11). The deterioration of cognitive function in patients with schizophrenia will lead to impairment of self-care, social, and occupational function (12). Therefore, the social impact of schizophrenia is very high. Current antipsychotics have limited, if any, efficacy for cognitive function.

The etiology of schizophrenia remains unclear. Oxidative stress and glutamate-related dysfunction, potentiating each other in a vicious circle, are interdependently involved in the pathogenesis of schizophrenia (13, 14). Adolescence or early adulthood is the critical period when schizophrenia typically arises, while glutamate is the main excitatory neurotransmitter that mediates puberty (15). Oxidative stress and genetic/environmental factors converge during neurodevelopment, leading to the impairment of neural connectivity and synchronization, as well as to cognitive deficits in early psychosis patients (16).

This review highlights a recent development surrounding N-methyl-D-aspartate receptor (NMDAR) modulators and antioxidants, paving the way for biomarker guided early detection and intervention of high-risk individuals (17).

## Importance of Early Detection and Intervention of Schizophrenia

Most individuals experience a period of prodromal symptoms prior to the diagnosis of schizophrenia (18). Before full-blown psychotic symptoms appear, individuals may experience changes in cognition, behavior, and function (19). Therefore, it is crucial to identify populations at high risk of schizophrenia to initiate early intervention (20). Improved diagnostic tools, the advent of atypical antipsychotic and the development of phase-specific psychosocial treatments have made intervention research in people at prodrome or ultra-high risk or people with attenuated psychosis syndrome for developing schizophrenia possible (21).

Antipsychotic medications, however, have not yet been approved for such populations, mainly because prolonged exposure to antipsychotic medication has been associated with various side effects such as weight gain, metabolic syndrome and hyperlipidemia (22, 23). First-generation antipsychotics, which block the majority of D2 dopamine receptors in the putamen (24, 25), mainly exert effects on positive symptoms and generate numerous intolerable side effects such as parkinsonism (including tremor, rigidity, bradykinesia), akathisia, dystonia, and prolactinemia (26). Newer atypical antipsychotics targeting both dopamine D2 and serotonin 5HT2 receptors (24, 26, 27) have been suggested to be superior to conventional agents in terms of efficacy for positive symptoms and perhaps negative symptoms (28–30). Despite this, there were a considerable percentage of patients resistant or only partially responsive to available medications (31). Moreover, side-effect profiles of second-generation antipsychotics, including obesity, diabetes mellitus, hyperlipidemia, metabolic syndrome, and sudden cardiac death, limit their clinical use (32–34). A substantial portion of schizophrenia patients refuse or cannot tolerate antipsychotics due to poor response and/or side effects (24). Further, long-term antipsychotics use is associated with cognitive impairment (35).

Most prodromal patients receive no or very brief, if any, antipsychotic treatment, due to safety concerns (36). To date, there is neither approved medication for prodromal schizophrenia, nor reliable outcome predictor for its conversion to full-blown schizophrenia. Therefore, developing early diagnosis and intervention strategy is very important.

## The Glutamate Hypothesis of Schizophrenia

In addition to dopaminergic neurotransmission, glutamatergic neurotransmission has gained more attentions lately as the key deficit of schizophrenia (37–44). Glutamate has two major receptor families: (1) ionotropic receptors, consisting of N-methyl-D-aspartate (NMDA), α-amino-3-hydroxy-5-methyl-4-isoxazolepropionic acid (AMPA), and kainate receptor subtypes, and (2) metabotropic receptors (mGluRs), which are G-protein-coupled receptors.

While glutamatergic outputs appear widespread over the corticolimbic system, disinhibition of the glutamatergic output from the subiculum to the ventral tegmental area leads to the hyperdopaminergic state with treatment of NMDA receptor (NMDAR) antagonists (45).

## Hypofunction of NMDAR-mediated Neurotransmission in Schizophrenia

NMDAR, a heteromeric ion channel, formed from a number of subunits (NR1, NR2ANR2D, NR3A, and NR3B), plays an important role in neurocognition. NMDAR antagonists, such as phencyclidine (PCP) and ketamine, induce psychosis which resembles schizophrenia more closely than the amphetamine/dopamine agonist do (46). The former causes not only positive symptoms, but also negative symptoms and cognitive deficits associated with schizophrenia. Moreover, glycine transporter inhibitors could reverse ketamine-induced effects (37, 47–50). Decreases in NMDAR density were found in post-mortem tissue from schizophrenic patients (51). The above evidence suggests that NMDAR dysfunction may be a critical deficit in schizophrenia (40, 43, 44, 52). Modulation of NMDAR has been proposed as a possible therapy for schizophrenia, including its prodrome (26, 37, 48, 53–56).

## Abnormal Plasticity of AMPA and Kainate Receptors in Schizophrenia

While some glutamatergic synapses have only AMPA receptors (AMPARs) or only NMDARs, most have both receptors. NMDAR modulators may regulate not only NMDARs but also AMPARs (57). Similar to NMDARs, AMPARs modulate fast glutamate transmission, neuronal circuit remodeling and higher order cognitive functions such as learning and memory; and abnormalities of AMPAR trafficking contribute to dysfunction in brain diseases such as schizophrenia (58). AMPAR subunits (GluR1-4) assemble to form AMPAR complexes in the lumen of the endoplasmic reticulum. Recently, the possibility of AMPAR dysfunction has been proposed to explain abnormalities in glutamate neurotransmission associated with the pathophysiology of schizophrenia (59). Topiramate, an antiepileptic drug with AMPAR antagonist activity has been demonstrated to improve schizophrenia as an adjunctive therapy; however, its efficacy may occur via GABA neurotransmission, as AMPAR antagonism occurs only at high concentrations (60, 61). Beneficial effects of CX516 and minocycline on cognitive domains appeared insignificant with rigorous statistical analyses (62). Newer AMPAR modulators such as UoS12258 which may possess precognitive properties deserve further studies (63).

Studies of kainate receptors (KARs) met difficulties because of the lack of specific activators or blockers for the receptors. First, kainate can also activate AMPARs. Second, AMPA, activates many KARs too (64).

## Role of the mGluR Allosteric Modulation in Schizophrenia

The mGluRs, consisting of eight subtypes, provide a wide range of targets to modulate NMDAR function as well as glutamate release. Preclinical studies demonstrated that activation of the mGluR2/3 down-regulated the excessive dopamine release caused by treatment with NMDAR antagonists (65). A clinical trial showed that an mGluR2/3 agonist, which down-regulates disinhibited glutamate release, exhibited antipsychotic properties (66). There have also been advances in the discovery of highly selective positive allosteric modulators (PAMs) of mGluR2 and mGluR5 for the treatment of schizophrenia (67). The mGluR5 PAMs counter aberrant neuronal activity generated by NMDAR antagonists in the prefrontal cortex (68). Recently, more subtype-selective allosteric modulators for various mGluRs instill hopes of better or alternative treatments for (subgroups of) schizophrenia (69).

## Oxidative Stress in Schizophrenia

Current evidence supports that increased oxidative stress-induced cellular damage of macromolecules may play a role in schizophrenia, and schizophrenia patients have abnormal antioxidant defenses as observed in their peripheral blood (70–72), CSF (73), and postmortem brain tissues (74, 75). Evidence from genetic studies also suggests that schizophrenia patients may have a reduced ability to mount an adequate antioxidant defense (76).

The failure of antioxidant defenses to protect against free-radical generation damages cell membranes, impacts on neurotransmission and, ultimately, leads to phenotypes of schizophrenia (75). Important free radicals include hydrogen peroxide, the hydroxyl radical, nitric oxide (NO), and the superoxide radical. In the rate-limiting step of purine catabolism, xanthine oxidase catalyzes the conversion of xanthine to uric acid, an important antioxidant, and generates superoxide radicals. Superoxide dismutase catalyzes the conversion of superoxide radicals to hydrogen peroxide. Both catalase and glutathione peroxidase converts hydrogen peroxide to water and oxygen. Reduced glutathione is oxidized by glutathione peroxidase to oxidized glutathione. Glutathione peroxidase also converts nitrate (a by-product of NO radicals) to nitrite. Nitrite is often used as a marker for NO activity. Hydroxyl radicals, produced from both hydrogen peroxide and NO, promote apoptosis, DNA damage, protein carbonylation, and lipid peroxidation. Vitamin E, also acting as an antioxidant, can inhibit lipid peroxidation. Thiobarbituric acid reactive substances (TBARS) and malondialdehyde (MDA) are important end products of lipid peroxidation (77).

## Modulation of Oxidative Stress in Patients with Schizophrenia

Clinical trials also support an association between oxidative stress and schizophrenia. Treatment with the antioxidant N-acetylcysteine significantly reduced psychopathology in schizophrenia (78). Nevertheless, N-acetylcysteine may not represent an optimal antioxidant therapy, as its principal modus operandi, the supply of increased cysteine for glutathione biosynthesis, is of limited help unless the brain can use it to produce, recycle and utilize glutathione (13). Another important study also found that supplementation with fish oil significantly reduced the progression to first-episode psychosis in subjects with prodromal symptoms (79). However, many subjects in the study also carried severe depressive symptoms, hampering the conclusion of the study. Anyhow, these findings suggest that oxidative stress levels may be a biomarker of schizophrenia risk and response to adjunctive antioxidant treatment.

## Linking Oxidative Stress and NMDAR Hypofunction in Schizophrenia Pathogenesis

Molecular, genetic and pathological evidence suggests that not only oxidative stress but also NMDAR hypofunction contribute to schizophrenia pathophysiology. Evidence now suggests that these factors are mechanistically interdependent and contribute to a common schizophrenia-associated pathology (13, 14).

There are clear similarities between the impact of developmental NMDAR hypofunction and that of oxidative stress on the adult rodent: both cause similar behavioral and cognitive disturbances. Increasing evidence suggests that NMDAR hypofunction and oxidative stress may be reciprocally linked (13, 14, 80). The NMDAR is regulated by redox state: both GRIN1 and GRIN2A possess pairs of reduction-oxidation reaction (redox)-sensitive cysteine residues whose disulfide bond formation decreases NMDAR currents (80), while an overlapping group of cysteine residues are subject to inhibitory S-nitrosylation, which facilitates disulfide bond formation (80, 81).

Recently, it has been shown that changes in intracellular redox status can also modulate NMDAR activity in a manner that is relevant to age-dependent cognitive decline (82). Age-associated shifts in intracellular redox state to a pro-oxidizing environment have been linked to reduced NMDAR activity via the redox regulation of calcium/calmodulin-dependent protein kinase type II (CaMKII), and can be rescued by intracellular glutathione (83).

Whether NMDAR-related dysfunction may influence the modulation of oxidative stress and whether the modulation of oxidative stress can alter NMDAR-related neurotransmission both also deserve further studies.

## Searching for Diagnostic and Therapeutic Biomarkers of Schizophrenia

At present, the diagnosis and treatment response of schizophrenia rely on clinical manifestation. There have been lots of post-mortem brain studies (84). However, RNA expressions may be affected by many factors under post-mortem condition. Therefore, it's needed to establish peripheral, accessible biological markers for mental illness (85). Lymphocytes or white cells have been suggested to be a neural probe because numerous studies showed similarities between receptor expression and mechanisms of transduction processes of cells in the nervous system (e.g., neurons and glia) and lymphocytes (86). Blood-derived RNA has become a convenient alternative to traditional tissue biopsy-derived RNA (87).

Several potential markers have been reported. Hashimoto et al reported that serum levels of D-serine were lower in schizophrenic patients than in healthy subjects (88). Besides, the expression of apolipoprotein D was increased in the plasma and brains of individuals with schizophrenia (89). S100B is a calcium-binding protein produced by astroglial cells. It has also been reported that schizophrenic patients, compared with healthy subjects, have higher DRD3 mRNA levels (85) and lower AKT1 protein levels (90) in peripheral lymphocytes. Adrenomedullin mRNA levels in lymphoblastoid cell lines of male schizophrenia patients was higher than in controls (91). Via microarray technique, six genes were suggested to be biomarkers of schizophrenia (92). Another study demonstrated that mRNA expression of eight biomarkers could be discriminated between schizophrenia, bipolar disorder, and controls (87). However, developing more suitable biomarkers for schizophrenia in future studies is warranted because there exists a large overlap between patients and controls in present biomarker studies.

## NMDAR- and Oxidative-Related Biomarkers of Schizophrenia

NMDAR-related markers are scanty. Lin et al found that the G72 (D-amino acid oxidase activator, DAOA) protein level in plasma was much higher in patients with schizophrenia than in healthy controls (93). G72, functioning as a D-amino acid oxidase (DAAO) activator (DAOA), exists exclusively in 4 primates including humans. The study suggests that peripheral G72 concentration may be characteristic of schizophrenia. The finding has been replicated independently (94). G72 is a huge protein. Its longest protein is called LG72 and consists of 153 amino acids. Its complex interactions deserve intensive study to elucidate the pathogenesis and pathophysiology of schizophrenia (95). Liquid chromatography-mass spectrometry (LC-MS)-based proteomics and metabolomics that have been used to discover protein and metabolite markers in clinical diseases may be helpful to elucidate the function of G72 and its interaction with other proteins.

A previous study also found that mRNA expression levels of *SLC7A11* and *SLC3A2* were lower in patients with schizophrenia than healthy individuals (96). SLC3A2 and SLC7A11 are two subunits of the cystine/glutamate antiporter system $x_{\text{c}}^{-}$ which plays a critical role in the regulation of glutamate release. DAAO is responsible for degrading D-serine and other D-amino acids (97). A recent study found that its level in peripheral blood was higher with cognitive aging (98). Serine hydroxyl-methyltransferase 2 (SHMT2) is an isoenzyme that catalyzes the reversible conversion of serine and tetrahydrofolate (THF) to glycine and methylene THF. Phosphoserine aminotransferase 1 (PSAT1) is required for the phosphorylated pathway of L-serine biosynthesis. Uptake of D-serine and L-serine into neurons and astrocytes is predominantly mediated by the serine transporter (ASCT1) subtype. The aforementioned genes/proteins that can regulate glutamate release and NMDAR function may be implicated in the pathogenesis of schizophrenia. Further, a recent study suggests that altered NMDAR signaling and parameters may have the potential to be used to detect vulnerability toward schizophrenia in individuals early in the disease process and thereby enable early intervention in a subgroup of patients (17).

Patients with schizophrenia also exhibit abnormal blood oxidative stress parameters, including total antioxidant status, glutathione peroxidase, catalase, superoxide dismutase, and nitrite (71, 77). It has been suggested that oxidative stress may serve as a potential biomarker in the etiopathophysiology, clinical course (including predicting conversion of high-risk symptoms to psychosis), symptomatology, cognitive function, and treatment response by antioxidants in patients with schizophrenia (16, 77, 99–101).

## Mismatch Negativity as an Objective Measurement for NMDA Function and a Biomarker for Schizophrenia

Mismatch negativity (MMN) has been proven to be related to NMDAR and has been shown to be reduced in schizophrenia. Previous studies have successfully established a method to generate reliable MMNs and have demonstrated the involvement of the NMDAR in the genesis of MMN (102, 103). Computational model was created to explain the observed functional MRI (fMRI) time-series data by using a state-space model (104), and has been used to model the evoked components as measured by electroencephalography (EEG) or magnetoencephalography (MEG), that has been used to study the production mechanisms of MMN and P300 (103).

Building a computational model for MMN may be helpful for exploring the network of MMN in schizophrenia and its treatment by the NMDAR enhancers such as D-serine (105). Longitudinal studies have also shown that MMN recordings can assist in predicting the conversion from the prodromal phase to psychosis (106).

## DAAO Inhibition for Schizophrenia

D-serine is more potent than other NMDAR co-agonists as the neurotransmitter for the glycine-site of the NMDAR (107). DAAO, a flavoenzyme of peroxisomes existing in the brain, kidney and liver of mammals, is responsible for degrading D-serine, D-alanine, and other D-amino acids. Therefore, one of the avenues to enhance NMDAR function is via inhibiting DAAO activity.

Sodium benzoate, a DAAO inhibitor, can elevate synaptic concentrations of D-amino acids, like D-serine and D-alanine, and thereby enhance NMDA neurotransmission. Previous clinical trials have studied the potential of sodium benzoate as an adjuvant therapy for schizophrenia. The first clinical trial suggested that sodium benzoate is beneficial in improving the clinical symptoms including positive and negative symptoms, cognitive and global functioning and quality of life in patients with chronic schizophrenia (40). The effect size of sodium benzoate treatment for Positive and Negative Syndrome Scale (PANSS) total score from baseline to endpoint was 1.76, which was much higher than the effect size (0.51) of sarcosine adjuvant therapy for the PANSS total score in patients with chronic schizophrenia (108).

## Glutamatergic Modulators in Patients with Persistent Psychotic Symptoms

Only a minority of patients with first-onset schizophrenia return to their original level of functioning. Among individuals who respond poorly to antipsychotics (which are principally dopamine antagonists), their glutamatergic/NMDAR dysfunction may lead to failures by the treatment. While second- and third-generation antipsychotics are increasingly used, therapy for refractory schizophrenia remains a great challenge. Even with the treatment of clozapine (the last-line therapy for schizophrenia), a substantial portion of patients still suffer from persistent psychotic symptoms. However, after many clinical trials with various agents, including diverse glutamatergic modulators, there is no convincing evidence to demonstrate the efficacy of adjuvant therapy for clozapine-resistant patients (109). In a recent study, sodium benzoate even showed a beneficial effect on positive and negative symptoms and quality of life with the dose of 2 g/day in patients with clozapine-resistant schizophrenia (43).

## Strategies of Early Detection and Intervention of Prodromal Schizophrenia

The prodromal phase of schizophrenic disorders has been recognized since the Nineteenth century (110). Recently, the Criteria of Prodromal Syndromes (COPS) diagnostic criteria have been applied; there are three operationally defined prodromal syndromes: attenuated positive psychotic symptom syndrome, brief intermittent psychotic syndrome, and genetic risk and recent functional decline syndrome (18, 111, 112). The PRIME prodromal research team in Yale University has also developed a semi-structured interview called the Structured Interview for Prodromal syndromes (SIPS) (113). The SIPS is utilized to rate presenting symptomatology and to determine if COPS criteria are met. The Scale of Prodromal Symptoms (SOPS) (114), embedded in SIPS, is a 19-item scale designed to measure the severity of prodromal symptoms. The SOPS contains four subscales: five positive, six negative, four disorganization, and four general symptom items. The detection and intervention of young people in the prodromal phase is a newly developed area in psychiatry (115), and the ethical considerations about treatment options must be treated with sensitivity (116).

Standard guidelines have been used in our previous studies aiming to establish or examine prodromal or ultra-high-risk (UHR) (112), clinical high risk (117), and 5 at-risk mental state (118). Recently, objective strategies have been emphasized for screening prodromal illness in many studies. The fMRI with magnetic resonance spectroscopy (MRS) is one of those that identify early stage of mental illness. Individuals with prodromal symptoms demonstrated smaller differential activation in frontal regions in fMRI data (119).

The possibility of treatment intervention during the prodromal phase has a history almost as long as it was first identified (120). Both typical and atypical antipsychotics, including risperidone and olanzapine, have been utilized to reduce prodromal symptoms or the risk of progression to schizophrenia (121–123). However, safety and side effect concerns exist; and it remains unclear whether the benefits of antipsychotic treatments outweigh the risks (116).

Therefore, there is an urgent need to develop safer interventions for schizophrenic prodrome. D-serine (124) and fish oil (79) have been demonstrated to be beneficial as treatment of prodromal schizophrenia. Other antioxidants such as glucoraphanin have also shown potential in preventing the onset of psychosis in the adult offspring after maternal immune activation (125). Future trials with glutamate modulators or antioxidants in early psychosis and even prodromal schizophrenia should consider biomarker-guided treatment (16).

## Summary

It is generally recognized that intervention of early psychosis and prevention the progression of schizophrenic prodrome to full-blown schizophrenia is essential, in order to avoid subsequent functional deterioration. Current antipsychotic medications have not yet been approved for such populations mainly due to the lack of overt efficacy and various side effects including metabolic syndrome and hyperprolactinemia. Therefore, developing novel antipsychotic drugs with better efficacy and safety is critical. Compounds that can enhance the NMDAR have shown encouraging efficacies with favorable safety profiles in clinical trials for patients with schizophrenia. It will be valuable to test whether NMDAR enhancers are beneficial for patients with earlier phases of psychosis.

Identifying high risk populations who are prone to develop full-blown psychosis would be very helpful to apply early an intervention strategy to those people who are in need. It is important to search for biomarkers representing the pathophysiology of schizophrenia and more importantly, the biological changes in the process of early psychosis. In addition to dopamine hypothesis, dysfunction of glutamate signaling, and dysregulation of oxidative stress have been considered to play important roles in early psychosis and schizophrenic prodrome. It will be interesting to search for potential biomarkers that are related to glutamate and oxidative stress modulations via blood-based or brain imaging approaches.

Combining biomarkers and novel drug development for early psychosis is critical in future studies. Notably, the intervention that can both treat early psychosis and serve as the biomarker might have more potential to reach the goal.

## Author Contributions

All authors listed have made a substantial, direct and intellectual contribution to the work, and approved it for publication.

### Conflict of Interest Statement

The authors declare that the research was conducted in the absence of any commercial or financial relationships that could be construed as a potential conflict of interest.

## References

[1] Green MF. What are the functional consequences of neurocognitive deficits in schizophrenia? Am J Psychiatry. (1996) 153:321–30. 10.1176/ajp.153.3.321 8610818

[2] Heaton RK Gladsjo JA Palmer BW Kuck J Marcotte TD Jeste DV. Stability and course of neuropsychological deficits in schizophrenia. Arch Gen Psychiatry. (2001) 58:24–32. 10.1001/archpsyc.58.1.24 11146755

[3] Lien YJ Tsuang HC Chiang A Liu CM Hsieh MH Hwang TJ. The multidimensionality of schizotypy in nonpsychotic relatives of patients with schizophrenia and its applications in ordered subsets linkage analysis of schizophrenia. Am J Med Genet B Neuropsychiatr Genet. (2010) 153B:1–9. 10.1002/ajmg.b.30948 19326390

[4] Tsuang MT Stone WS Faraone SV. Understanding predisposition to schizophrenia: toward intervention and prevention. Can J Psychiatry. (2002) 47:518–26. 10.1177/070674370204700603 12211879

[5] Chen WJ Chang CH Liu SK Hwang TJ Hwu HG. Sustained attention deficits in nonpsychotic relatives of schizophrenic patients: a recurrence risk ratio analysis. Biol Psychiatry. (2004) 55:995–1000. 10.1016/j.biopsych.2004.01.010 15121483

[6] Chen WJ Liu SK Chang CJ Lien YJ Chang YH Hwu HG. Sustained attention deficit and schizotypal personality features in nonpsychotic relatives of schizophrenic patients. Am J Psychiatry. (1998) 155:1214–20. 10.1176/ajp.155.9.1214 9734545

[7] Holden C Neuroscience. Deconstruct Schizophr Sci. (2003) 299:333–5. 10.1126/science.299.5605.333

[8] Liu SK Chen WJ Chang CJ Lin HN Effects of atypical neuroleptics on sustained attention deficits in schizophrenia: a trial of risperidone vs. haloperidol. Neuropsychopharmacology. (2000) 22:311–9. 10.1016/S0893-133X(99)00137-2 10693159

[9] Tsuang HC Lin SH Liu SK Hsieh MH Hwang TJ Liu CM. More severe sustained attention deficits in nonpsychotic siblings of multiplex schizophrenia families than in those of simplex ones. Schizophr Res. (2006) 87:172–80. 10.1016/j.schres.2006.03.045 16737801

[10] Pu S Nakagome K Itakura M Iwata M Nagata I Kaneko K. The association between cognitive deficits and prefrontal hemodynamic responses during performance of working memory task in patients with schizophrenia. Schizophr Res. (2016) 172:114–22. 10.1016/j.schres.2016.01.045 26830318

[11] Wright S Kochunov P Chiappelli J McMahon R Muellerklein F Wijtenburg SA. Accelerated white matter aging in schizophrenia: role of white matter blood perfusion. Neurobiol Aging. (2014) 35:2411–8. 10.1016/j.neurobiolaging.2014.02.016 24680326 PMC4087059

[12] Friedman JI Harvey PD Kemether E Byne W Davis KL. Cognitive and functional changes with aging in schizophrenia. Biol Psychiatry. (1999) 46:921–8. 10.1016/S0006-3223(99)00080-3 10509175

[13] Hardingham GE Do KQ. Linking early-life NMDAR hypofunction and oxidative stress in schizophrenia pathogenesis. Nat Rev Neurosci. (2016) 17:125–34. 10.1038/nrn.2015.19 26763624

[14] Steullet P Cabungcal JH Monin A Dwir D O'Donnell P Cuenod M. Redox dysregulation, neuroinflammation, and NMDA receptor hypofunction: a “central hub” in schizophrenia pathophysiology? Schizophr Res. (2016) 176:41–51. 10.1016/j.schres.2014.06.021 25000913 PMC4282982

[15] Giuliani FA Escudero C Casas S Bazzocchini V Yunes R Laconi MR. Allopregnanolone and puberty: modulatory effect on glutamate and GABA release and expression of 3alpha-hydroxysteroid oxidoreductase in the hypothalamus of female rats. Neuroscience. (2013) 243:64–75 10.1016/j.neuroscience.2013.03.053 23562943

[16] Conus P Seidman LJ Fournier M Xin L Cleusix M Baumann PS. N-acetylcysteine in a double-blind randomized placebo-controlled trial: toward biomarker-guided treatment in early psychosis. Schizophr Bull. (2018) 44:317–27. 10.1093/schbul/sbx093 29462456 PMC5815074

[17] Gunduz-Bruce H Kenney J Changlani S Peixoto A Gueorguieva R Leone C. A translational approach for NMDA receptor profiling as a vulnerability biomarker for depression and schizophrenia. Exp Physiol. (2017) 102:587–97. 10.1113/EP086212 28294453

[18] Yung AR McGorry PD McFarlane CA Jackson HJ Patton GC Rakkar A. Monitoring and care of young people at incipient risk of psychosis. Schizophr Bull. (1996) 22:283–303. 10.1093/schbul/22.2.283 8782287

[19] Yung AR Stanford C Cosgrave E Killackey E Phillips L Nelson B. Testing the ultra high risk (prodromal) criteria for the prediction of psychosis in a clinical sample of young people. Schizophr Res. (2006) 84:57–66. 10.1016/j.schres.2006.03.014 16630707

[20] Brewer WJ Wood SJ Phillips LJ Francey SM Pantelis C Yung AR. Generalized and specific cognitive performance in clinical high-risk cohorts: a review highlighting potential vulnerability markers for psychosis. Schizophr Bull. (2006) 32:538–55. 10.1093/schbul/sbj077 16782759 PMC2632242

[21] Phillips LJ Yung AR Yuen HP Pantelis C McGorry PD. Prediction and prevention of transition to psychosis in young people at incipient risk for schizophrenia. Am J Med Genet. (2002) 114:929–37. 10.1002/ajmg.b.10790 12457389

[22] Datta SS Kumar A Wright SD Furtado VA Russell PS. Evidence base for using atypical antipsychotics for psychosis in adolescents. Schizophr Bull. (2014) 40:252–4. 10.1093/schbul/sbt196 24361758 PMC3932092

[23] Kumar A Datta SS Wright SD Furtado VA Russell PS Atypical antipsychotics for psychosis in adolescents. Cochr Database Syst Rev. (2013) 62:1196–204. 10.1002/14651858.CD009582.pub2 PMC11339588 24129841

[24] Abbott A. Schizophrenia: the drug deadlock. Nature. (2010) 468:158–9. 10.1038/468158a 21068804

[25] Farde L Hall H Ehrin E Sedvall G. Quantitative analysis of D2 dopamine receptor binding in the living human brain by PET. Science. (1986) 231:258–61. 2867601 10.1126/science.2867601

[26] Insel TR. Rethinking schizophrenia. Nature. (2010) 468:187–93. 10.1038/nature09552 21068826

[27] Kapur S Zipursky RB Remington G. Clinical and theoretical implications of 5-HT2 and D2 receptor occupancy of clozapine, risperidone, and olanzapine in schizophrenia. Am J Psychiatry. (1999) 156:286–93. 9989565 10.1176/ajp.156.2.286

[28] Green MF Marshall BDJr Wirshing WC Ames D Marder SR McGurk S. Does risperidone improve verbal working memory in treatment-resistant schizophrenia? Am J Psychiatry. (1997) 154:799–804. 10.1176/ajp.154.6.799 9167507

[29] Lane HY Liu CC Chang WH. Risperidone for exclusively negative symptoms. Am J Psychiatry. (1999) 156:335. 9989576 10.1176/ajp.156.2.335

[30] Leucht S Wahlbeck K Hamann J Kissling W New generation antipsychotics vs. low-potency conventional antipsychotics: a systematic review and meta-analysis. Lancet. (2003) 361:1581–9. 10.1016/S0140-6736(03)13306-5 12747876

[31] Lieberman JA Stroup TS McEvoy JP Swartz MS Rosenheck RA Perkins DO. Effectiveness of antipsychotic drugs in patients with chronic schizophrenia. N Engl J Med. (2005) 353:1209–23. 10.1056/NEJMoa051688 16172203

[32] Gaulin BD Markowitz JS Caley CF Nesbitt LA Dufresne RL. Clozapine-associated elevation in serum triglycerides. Am J Psychiatry. (1999) 156:1270–2. 10450273 10.1176/ajp.156.8.1270

[33] Lu ML Lane HY Lin SK Chen KP Chang WH. Adjunctive fluvoxamine inhibits clozapine-related weight gain and metabolic disturbances. J Clin Psychiatry. (2004) 65:766–71. 10.4088/JCP.v65n0607 15291653

[34] Ray WA Chung CP Murray KT Hall K Stein CM. Atypical antipsychotic drugs and the risk of sudden cardiac death. N Engl J Med. (2009) 360:225–35. 10.1056/NEJMoa0806994 19144938 PMC2713724

[35] Husa AP Rannikko I Moilanen J Haapea M Murray GK Barnett J (2014). Lifetime use of antipsychotic medication and its relation to change of verbal learning and memory in midlife schizophrenia - An observation 9-year follow-up study. Schizophr Res. (2014) 158:134–41. 10.1016/j.schres.2014.06.035 25034761

[36] Marshall M Rathbone J Early intervention for psychosis. Cochr Database Syst Rev. (2011) 8:2158 10.1002/14651858.CD004718 PMC4163966 21678345

[37] Javitt DC Balla A Sershen H Lajtha A AE bennett research award reversal of phencyclidine-induced effects by glycine and glycine transport inhibitors. Biol Psychiatry. (1999) 45:668–79. 10.1016/S0006-3223(98)00237-6 10187996

[38] Lane HY Chang YC Liu YC Chiu CC Tsai GE. Sarcosine or D-serine add-on treatment for acute exacerbation of schizophrenia: a randomized, double-blind, placebo-controlled study. Arch Gen Psychiatry. (2005) 62:1196–204. 10.1001/archpsyc.62.11.1196 16275807

[39] Lane HY Huang CL Wu PL Liu YC Chang YC Lin PY. Glycine transporter I inhibitor, N-methylglycine (sarcosine), added to clozapine for the treatment of schizophrenia. Biol Psychiatry. (2006) 60:645–9. 10.1016/j.biopsych.2006.04.005 16780811

[40] Lane HY Lin CH Green MF Hellemann G Huang CC Chen PW. Add-on treatment of benzoate for schizophrenia: a randomized, double-blind, placebo-controlled trial of D-amino acid oxidase inhibitor. JAMA Psychiatry. (2013) 70:1267–75. 10.1001/jamapsychiatry.2013.2159 24089054

[41] Lane HY Lin CH Huang YJ Liao CH Chang YC Tsai GE. A randomized, double-blind, placebo-controlled comparison study of sarcosine (N-methylglycine) and D-serine add-on treatment for schizophrenia. Int J Neuropsychopharmacol. (2010) 13:451–60. 10.1017/S1461145709990939 19887019

[42] Lane HY Liu YC Huang CL Chang YC Liau CH Perng CH. Sarcosine (N-methylglycine) treatment for acute schizophrenia: a randomized, double-blind study. Biol Psychiatry. (2008) 63:9–12. 10.1016/j.biopsych.2007.04.038 17659263

[43] Lin CH Chang YC Huang YJ Chen PW Yang HT Lane HY. Sodium benzoate, a D-amino acid oxidase inhibitor, added to clozapine for the treatment of schizophrenia: a randomized, double-blind, placebo-controlled trial. Biol Psychiatry. (2018) 84:422–32. 10.1016/j.biopsych.2017.12.006 29397899

[44] Olney JW Farber NB. Glutamate receptor dysfunction and schizophrenia. Arch Gen Psychiatry. (1995) 52:998–1007. 10.1001/archpsyc.1995.03950240016004 7492260

[45] Lisman JE Coyle JT Green RW Javitt DC Benes FM Heckers S. Circuit-based framework for understanding neurotransmitter and risk gene interactions in schizophrenia. Trends Neurosci. (2008) 31:234–42. 10.1016/j.tins.2008.02.005 18395805 PMC2680493

[46] Krystal JH Karper LP Seibyl JP Freeman GK Delaney R Bremner JD. Subanesthetic effects of the noncompetitive NMDA antagonist, ketamine, in humans. Psychotomimetic, perceptual, cognitive, and neuroendocrine responses. Arch Gen Psychiatry. (1994) 51:199–214. 10.1001/archpsyc.1994.03950030035004 8122957

[47] Buchanan RW Javitt DC Marder SR Schooler NR Gold JM McMahon RP. The cognitive and negative symptoms in schizophrenia trial (CONSIST): the efficacy of glutamatergic agents for negative symptoms and cognitive impairments. Am J Psychiatry. (2007) 164:1593–602. 10.1176/appi.ajp.2007.06081358 17898352

[48] Chang HJ Lane HY Tsai GE. NMDA pathology and treatment of schizophrenia. Curr Pharm Des. (2014) 20:5118–26. 10.2174/1381612819666140110121908 24410561

[49] Javitt DC Balla A Burch S Suckow R Xie S Sershen H. Reversal of phencyclidine-induced dopaminergic dysregulation by N-methyl-D-aspartate receptor/glycine-site agonists. Neuropsychopharmacology (2004) 29:300–7. 10.1038/sj.npp.1300313 14560321

[50] Yang SY Hong CJ Huang YH Tsai SJ. The effects of glycine transporter I inhibitor, N-methylglycine (sarcosine), on ketamine-induced alterations in sensorimotor gating and regional brain c-Fos expression in rats. Neurosci Lett. (2010) 469:127–30. 10.1016/j.neulet.2009.11.058 19944746

[51] Sokolov BP. Expression of NMDAR1, GluR1, GluR7, and KA1 glutamate receptor mRNAs is decreased in frontal cortex of “neuroleptic-free” schizophrenics: evidence on reversible up-regulation by typical neuroleptics. J Neurochem. (1998) 71:2454–64. 10.1046/j.1471-4159.1998.71062454.x 9832144

[52] Coyle JT. The glutamatergic dysfunction hypothesis for schizophrenia. Harv Rev Psychiatry. (1996) 3:241–53. 10.3109/10673229609017192 9384954

[53] Hashimoto K Fujita Y Horio M Kunitachi S Iyo M Ferraris D. Co-administration of a D-amino acid oxidase inhibitor potentiates the efficacy of D-serine in attenuating prepulse inhibition deficits after administration of dizocilpine. Biol Psychiatry. (2009) 65:1103–6. 10.1016/j.biopsych.2009.01.002 19217074

[54] Heresco-Levy U Ermilov M Shimoni J Shapira B Silipo G Javitt DC Placebo-controlled trial of D-cycloserine added to conventional neuroleptics, olanzapine, or risperidone in schizophrenia. Am J Psychiatry. (2002) 159:480–2. 10.1176/appi.ajp.159.3.480 11870017

[55] Javitt DC. Glycine transport inhibitors and the treatment of schizophrenia. Biol Psychiatry. (2008) 63:6–8. 10.1016/j.biopsych.2007.09.017 18082555

[56] Javitt DC Zukin SR. Recent advances in the phencyclidine model of schizophrenia. Am J Psychiatry. (1991) 148:1301–8. 10.1176/ajp.148.10.1301 1654746

[57] Wei IH Chen KT Tsai MH Wu CH Lane HY Huang CC. Acute amino acid d-serine administration, similar to ketamine, produces antidepressant-like effects through identical mechanisms. J Agric Food Chem. (2017) 65:10792–803. 10.1021/acs.jafc.7b04217 29161812

[58] Kessels HW Malinow R. Synaptic AMPA receptor plasticity and behavior. Neuron. (2009) 61:340–50. 10.1016/j.neuron.2009.01.015 19217372 PMC3917551

[59] Tucholski J Simmons MS Pinner AL Haroutunian V McCullumsmith RE Meador-Woodruff JH. Abnormal N-linked glycosylation of cortical AMPA receptor subunits in schizophrenia. Schizophr Res. (2013) 146:177–83. 10.1016/j.schres.2013.01.031 23462048 PMC3655690

[60] Gibbs JW III Sombati S DeLorenzo RJ Coulter DA. Cellular actions of topiramate: blockade of kainate-evoked inward currents in cultured hippocampal neurons. Epilepsia. (2000) 41(Suppl. 243):10–16. 10.1111/j.1528-1157.2000.tb02164.x 10768293

[61] Tiihonen J Halonen P Wahlbeck K Repo-Tiihonen E Hyvarinen S Eronen M. Topiramate add-on in treatment-resistant schizophrenia: a randomized, double-blind, placebo-controlled, crossover trial. J Clin Psychiatry. (2005) 66:1012–5. 10.4088/JCP.v66n0808 16086616

[62] Iwata Y Nakajima S Suzuki T Keefe RS Plitman E Chung JK. Effects of glutamate positive modulators on cognitive deficits in schizophrenia: a systematic review and meta-analysis of double-blind randomized controlled trials. Mol Psychiatry. (2015) 20:1151–60. 10.1038/mp.2015.68 26077694 PMC5323255

[63] Ward SE Beswick P Calcinaghi N Dawson LA Gartlon J Graziani F Pharmacological characterization of N-[(2S)-5-(6-fluoro-3-pyridinyl)-2, 3-dihydro-1H-inden-2-yl]-2-propanesulfonamide: a novel, clinical AMPA receptor positive allosteric modulator. Br J Pharmacol. (2017) 174:370–85. 10.1111/bph.13696 28009436 PMC5301047

[64] Lerma J Marques JM. Kainate receptors in health and disease. Neuron. (2013) 80:292–311. 10.1016/j.neuron.2013.09.045 24139035

[65] Fell MJ Svensson KA Johnson BG Schoepp DD Evidence for the role of metabotropic glutamate (mGlu)2 not mGlu3 receptors in the preclinical antipsychotic pharmacology of the mGlu2/3 receptor agonist (-)-(1R,4S,5S,6S)-4-amino-2-sulfonylbicyclo[3.1.0]hexane-4,6-dicarboxylic acid (LY404039). J Pharmacol Exp Ther. (2008) 326:209–17. 10.1124/jpet.108.136861 18424625

[66] Patil ST Zhang L Martenyi F Lowe SL Jackson KA Andreev BV Activation of mGlu2/3 receptors as a new approach to treat schizophrenia: a randomized Ph 2 clinical trial. Nat Med. (2007) 13:1102–7. 10.1038/nm1632 17767166

[67] Conn PJ Lindsley CW Jones CK. Activation of metabotropic glutamate receptors as a novel approach for the treatment of schizophrenia. Trends Pharmacol Sci. (2009) 30:25–31. 10.1016/j.tips.2008.10.006 19058862 PMC2907735

[68] Lecourtier L Homayoun H Tamagnan G Moghaddam B Positive allosteric modulation of metabotropic glutam. Biol Psychiatry. (2007)62:739–46. 10.1016/j.biopsych.2006.12.003 17511968 PMC2910402

[69] Maksymetz J Moran SP Conn PJ. Targeting metabotropic glutamate receptors for novel treatments of schizophrenia. Mol Brain. (2017) 10:15. 10.1186/s13041-017-0293-z 28446243 PMC5405554

[70] Michelson AM. Biological role of the superoxide anion radical and of superoxyde-dismutase in cellular metabolism. C R Seances Soc Biol Fil. (1976) 170:1137–46. 193619

[71] Okusaga OO. Accelerated aging in schizophrenia patients: the potential role of oxidative stress. Aging Dis. (2014) 5:256–62. 10.14336/AD.2014.0500256 25110609 PMC4113515

[72] Sirota P Gavrieli R Wolach B. Overproduction of neutrophil radical oxygen species correlates with negative symptoms in schizophrenic patients: parallel studies on neutrophil chemotaxis, superoxide production and bactericidal activity. Psychiatry Res. (2003) 121:123–32. 10.1016/S0165-1781(03)00222-1 14656447

[73] Do KQ Trabesinger AH Kirsten-Kruger M Lauer CJ Dydak U Hell D. Schizophrenia: glutathione deficit in cerebrospinal fluid and prefrontal cortex *in vivo*. Eur J Neurosci. (2000) 12:3721–8. 10.1046/j.1460-9568.2000.00229.x 11029642

[74] Do KQ Cabungcal JH Frank A Steullet P Cuenod M. Redox dysregulation, neurodevelopment, and schizophrenia. Curr Opin Neurobiol. (2009) 19:220–30. 10.1016/j.conb.2009.05.001 19481443

[75] Yao JK Leonard S Reddy RD. Increased nitric oxide radicals in postmortem brain from patients with schizophrenia. Schizophr Bull. (2004) 30:923–34. 10.1093/oxfordjournals.schbul.a007142 15954198

[76] Chowdari KV Bamne MN Nimgaonkar VL. Genetic association studies of antioxidant pathway genes and schizophrenia. Antioxid Redox Signal. (2011) 15:2037–45. 10.1089/ars.2010.3508 20673164 PMC3159115

[77] Flatow J Buckley P Miller BJ. Meta-analysis of oxidative stress in schizophrenia. Biol Psychiatry. (2013) 74:400–9. 10.1016/j.biopsych.2013.03.018 23683390 PMC4018767

[78] Berk M Copolov D Dean O Lu K Jeavons S Schapkaitz I. N-acetyl cysteine as a glutathione precursor for schizophrenia–a double-blind, randomized, placebo-controlled trial. Biol Psychiatry. (2008) 64:361–8. 10.1016/j.biopsych.2008.03.004 18436195

[79] Amminger GP Schafer MR Papageorgiou K Klier CM Cotton SM Harrigan SM. Long-chain omega-3 fatty acids for indicated prevention of psychotic disorders: a randomized, placebo-controlled trial. Arch Gen Psychiatry. (2010) 67:146–54. 10.1001/archgenpsychiatry.2009.192 20124114

[80] Lipton SA Choi YB Takahashi H Zhang D Li W Godzik A. Cysteine regulation of protein function–as exemplified by NMDA-receptor modulation. Trends Neurosci. (2002) 25:474–80. 10.1016/S0166-2236(02)02245-2 12183209

[81] Choi YB Lipton SA. Identification and mechanism of action of two histidine residues underlying high-affinity Zn^2+^ inhibition of the NMDA receptor. Neuron. (1999) 23:171–80. 10.1016/S0896-6273(00)80763-1 10402203

[82] Guidi M Kumar A Foster TC. Impaired attention and synaptic senescence of the prefrontal cortex involves redox regulation of NMDA receptors. J Neurosci. (2015) 35:3966–77. 10.1523/JNEUROSCI.3523-14.2015 25740525 PMC4348191

[83] Bodhinathan K Kumar A Foster TC. Intracellular redox state alters NMDA receptor response during aging through Ca^2+^/calmodulin-dependent protein kinase II. J Neurosci. (2010) 30:1914–24. 10.1523/JNEUROSCI.5485-09.2010 20130200 PMC2853968

[84] Stewart RJ Chen B Dowlatshahi D MacQueen GM Young LT. Abnormalities in the cAMP signaling pathway in post-mortem brain tissue from the stanley neuropathology consortium. Brain Res Bull. (2001) 55:625–9. 10.1016/S0361-9230(01)00524-X 11576759

[85] Ilani T Ben-Shachar D Strous RD Mazor M Sheinkman A Kotler M. A peripheral marker for schizophrenia: increased levels of D3 dopamine receptor mRNA in blood lymphocytes. Proc Natl Acad Sci USA. (2001) 98:625–8. 10.1073/pnas.98.2.625 11149951 PMC14638

[86] Gladkevich A Kauffman HF Korf J. Lymphocytes as a neural probe: potential for studying psychiatric disorders. Prog Neuropsychopharmacol Biol Psychiatry. (2004) 28:559–76. 10.1016/j.pnpbp.2004.01.009 15093964

[87] Tsuang MT Nossova N Yager T Tsuang MM Guo SC Shyu KG. Assessing the validity of blood-based gene expression profiles for the classification of schizophrenia and bipolar disorder: a preliminary report. Am J Med Genet B Neuropsychiatr Genet. (2005) 133B:1–5. 10.1002/ajmg.b.30161 15645418

[88] Hashimoto K Fukushima T Shimizu E Komatsu N Watanabe H Shinoda N. Decreased serum levels of D-serine in patients with schizophrenia: evidence in support of the N-methyl-D-aspartate receptor hypofunction hypothesis of schizophrenia. Arch Gen Psychiatry. (2003) 60:572–6. 10.1001/archpsyc.60.6.572 12796220

[89] Thomas EA Copolov DL Sutcliffe JG. From pharmacotherapy to pathophysiology: emerging mechanisms of apolipoprotein D in psychiatric disorders. Curr Mol Med. (2003) 3:408–18. 10.2174/1566524033479681 12942994

[90] Emamian ES Hall D Birnbaum MJ Karayiorgou M Gogos JA. Convergent evidence for impaired AKT1-GSK3beta signaling in schizophrenia. Nat Genet. (2004) 36:131–7. 10.1038/ng1296 14745448

[91] Huang CH Chen ML Tsai YL Tsai MT Chen CH. Elevated adrenomedullin mRNA in lymphoblastoid cells from schizophrenic patients. Neuroreport. (2004) 15:1443–6. 10.1097/01.wnr.0000132202.69212.79 15194870

[92] Glatt SJ Everall IP Kremen WS Corbeil J Sasik R Khanlou N. Comparative gene expression analysis of blood and brain provides concurrent validation of SELENBP1 up-regulation in schizophrenia. Proc Natl Acad Sci USA. (2005) 102:15533–8. 10.1073/pnas.0507666102 16223876 PMC1266138

[93] Lin CH Chang HT Chen YJ Huang CH Tun R Tsai GE. Distinctively higher plasma G72 protein levels in patients with schizophrenia than in healthy individuals. Mol Psychiatry. (2014) 19:636–7. 10.1038/mp.2013.80 23857119

[94] Akyol ES Albayrak Y Aksoy N Sahin B Beyazyuz M Kuloglu M. Increased serum G72 protein levels in patients with schizophrenia: a potential candidate biomarker. Acta Neuropsychiatr. (2017) 29:80–6. 10.1017/neu.2016.34 27412497

[95] Chang SL Hsieh CH Chen YJ Wang CM Shih CS Huang PW. The C-terminal region of G72 increases D-amino acid oxidase activity. Int J Mol Sci. (2013) 15:29–43. 10.3390/ijms15010029 24362575 PMC3907796

[96] Lin CH Lin PP Lin CY Huang CH Huang YJ Lane HY. Decreased mRNA expression for the two subunits of system xc(-), SLC3A2 and SLC7A11, in WBC in patients with schizophrenia: evidence in support of the hypo-glutamatergic hypothesis of schizophrenia. J Psychiatr Res. (2016) 72:58–63. 10.1016/j.jpsychires.2015.10.007 26540405

[97] Vanoni MA Cosma A Mazzeo D Mattevi A Todone F Curti B. Limited proteolysis and X-ray crystallography reveal the origin of substrate specificity and of the rate-limiting product release during oxidation of D-amino acids catalyzed by mammalian D-amino acid oxidase. Biochemistry. (1997) 36:5624–32. 10.1021/bi963023s 9153402

[98] Lin CH Yang HT Chiu CC Lane HY. Blood levels of D-amino acid oxidase vs. D-amino acids in reflecting cognitive aging. Sci Reports. (2017) 7:14849. 10.1038/s41598-017-13951-7 29093468 PMC5665939

[99] Matsuzawa D Hashimoto K. Magnetic resonance spectroscopy study of the antioxidant defense system in schizophrenia. Antioxid Redox Signal. (2011) 15:2057–65. 10.1089/ars.2010.3453 20712400

[100] Perkins DO Jeffries CD Addington J Bearden CE Cadenhead KS Cannon TD Toward a psychosis risk blood diagnostic for persons experiencing high-risk symptoms: preliminary results from the NAPLS project. Schizophr Bull. (2015) 41:419–28. 10.1093/schbul/sbu099 25103207 PMC4332942

[101] Wu JQ Chen DC Tan YL Tan S Wang Z Yang F. Association of altered CuZn superoxide dismutase and cognitive impairment in schizophrenia patients with tardive dyskinesia. J Psychiatr Res. (2014) 58:167–74. 10.1016/j.jpsychires.2014.07.028 25151339

[102] Chen JC Hammerer D D'Ostilio K Casula EP Marshall L Tsai CH. Bi-directional modulation of somatosensory mismatch negativity with transcranial direct current stimulation: an event related potential study. J Physiol. (2014) 592:745–57. 10.1113/jphysiol.2013.260331 24366257 PMC3934712

[103] Chen JC Hammerer D Strigaro G Liou LM Tsai CH Rothwell JC. Domain-specific suppression of auditory mismatch negativity with transcranial direct current stimulation. Clin Neurophysiol. (2014) 125:585–92. 10.1016/j.clinph.2013.08.007 24051072

[104] Friston KJ Harrison L Penny W. Dynamic causal modelling. Neuroimage. (2003) 19:1273–302. 10.1016/S1053-8119(03)00202-7 12948688

[105] Kantrowitz JT Epstein ML Lee M Lehrfeld N Nolan KA Shope C. Improvement in mismatch negativity generation during d-serine treatment in schizophrenia: correlation with symptoms. Schizophr Res. (2018) 191:70–9. 10.1016/j.schres.2017.02.027 28318835

[106] Light GA Naatanen R. Mismatch negativity is a breakthrough biomarker for understanding and treating psychotic disorders. Proc Natl Acad Sci USA. (2013) 110:15175–6. 10.1073/pnas.1313287110 23995447 PMC3780842

[107] Lin CH Lane HY Tsai GE. Glutamate signaling in the pathophysiology and therapy of schizophrenia. Pharmacol Biochem Behav. (2012) 100:665–77. 10.1016/j.pbb.2011.03.023 21463651

[108] Tsai G Lane HY Yang P Chong MY Lange N. Glycine transporter I inhibitor, N-methylglycine (sarcosine), added to antipsychotics for the treatment of schizophrenia. Biol Psychiatry. (2004) 55:452–6. 10.1016/j.biopsych.2003.09.012 15023571

[109] Harrison PJ. D-amino acid oxidase inhibition: a new glutamate twist for clozapine augmentation in schizophrenia? Biol Psychiatry. (2018) 84:396–8. 10.1016/j.biopsych.2018.06.001 30165950

[110] Bleuler E Dementia praecox or the group of schizophrenias. Vertex. (2010) 21:394–400. 21218204

[111] Woods SW Miller TJ McGlashan TH. The “prodromal” patient: both symptomatic and at-risk. CNS Spectr. (2001) 6:223–32. 10.1017/S1092852900008609 16951657

[112] Yung AR Phillips LJ Yuen HP Francey SM McFarlane CA Hallgren M. Psychosis prediction: 12-month follow up of a high-risk (“prodromal”) group. Schizophr Res. (2003) 60:21–32. 10.1016/S0920-9964(02)00167-6 12505135

[113] Miller TJ McGlashan TH Rosen JL Somjee L Markovich PJ Stein K. Prospective diagnosis of the initial prodrome for schizophrenia based on the Structured Interview for Prodromal Syndromes: preliminary evidence of interrater reliability and predictive validity. Am J Psychiatry. (2002) 159:863–5. 10.1176/appi.ajp.159.5.863 11986145

[114] Miller TJ McGlashan TH Woods SW Stein K Driesen N Corcoran CM Symptom assessment in schizophrenic prodromal states. The Psychiatric quarterly. Win. (1999) 70:273–87. 10.1023/a:1022034115078 10587984

[115] McGorry PD Yung AR Phillips LJ Yuen HP Francey S Cosgrave EM. Randomized controlled trial of interventions designed to reduce the risk of progression to first-episode psychosis in a clinical sample with subthreshold symptoms. Arch Gen Psychiatry. (2002) 59:921–8. 10.1001/archpsyc.59.10.921 12365879

[116] Phillips LJ McGorry PD Yung AR McGlashan TH Cornblatt B Klosterkotter J. Prepsychotic phase of schizophrenia and related disorders: recent progress and future opportunities. Br J Psychiatry Suppl. (2005) 48:s33–44. 10.1192/bjp.187.48.s33 16055805

[117] Addington J Heinssen R. Prediction and prevention of psychosis in youth at clinical high risk. Annu Rev Clin Psychol. (2012) 8:269–89. 10.1146/annurev-clinpsy-032511-143146 22224837

[118] Demjaha A Valmaggia L Stahl D Byrne M McGuire P. Disorganization/cognitive and negative symptom dimensions in the at-risk mental state predict subsequent transition to psychosis. Schizophr Bull. (2012) 38:351–9. 10.1093/schbul/sbq088 20705805 PMC3283152

[119] Gur RE Gur RC. Functional magnetic resonance imaging in schizophrenia. Dialog Clin Neurosci. (2010) 12:333–43. 20954429 10.31887/DCNS.2010.12.3/rgur PMC3181978

[120] Sullivan HS. The onset of schizophrenia (1927). Am J Psychiatry (1994) 151:134–9. 10.1176/ajp.151.6.134 8192188

[121] Falloon IR. Early intervention for first episodes of schizophrenia: a preliminary exploration. Psychiatry. (1992) 55:4–15. 10.1080/00332747.1992.11024572 1557469

[122] McGorry PD Warner R. Consensus on early intervention in schizophrenia. Schizophr Bull. (2002) 28:543–4. 10.1093/oxfordjournals.schbul.a006962 12645686

[123] Woods SW Breier A Zipursky RB Perkins DO Addington J Miller TJ Randomized trial of olanzapine vs. placebo in the symptomatic acute treatment of the schizophrenic prodrome. Biol Psychiatry. (2003) 54:453–64. 10.1016/S0006-3223(03)00321-4 12915290

[124] Kantrowitz JT Woods SW Petkova E Cornblatt B Corcoran CM Chen H. D-serine for the treatment of negative symptoms in individuals at clinical high risk of schizophrenia: a pilot, double-blind, placebo-controlled, randomised parallel group mechanistic proof-of-concept trial. Lancet Psychiatry. (2015) 2:403–12. 10.1016/S2215-0366(15)00098-X 26360284

[125] Matsuura A Ishima T Fujita Y Iwayama Y Hasegawa S Kawahara-Miki R. Dietary glucoraphanin prevents the onset of psychosis in the adult offspring after maternal immune activation. Sci Rep. (2018) 8:2158 10.1038/s41598-018-20538-3 29391571 PMC5794794

